# High-Temperature Liquid–Liquid Phase Transition in Glass-Forming Liquid Pd_43_Ni_20_Cu_27_P_10_

**DOI:** 10.3390/ma16124353

**Published:** 2023-06-13

**Authors:** Huanyi Zhou, Pengfei Yu, Xiaoyu Miao, Cunjin Peng, Lulu Fu, Conghui Si, Qifang Lu, Shunwei Chen, Xiujun Han

**Affiliations:** 1 School of Materials Science and Engineering, Qilu University of Technology (Shandong Academy of Sciences), Jinan 250353, China; 2 School of Materials Science and Engineering, Shanghai Jiao Tong University, Shanghai 200240, China

**Keywords:** liquid–liquid phase transition, Pd-Ni-Cu-P, endothermic, flash differential scanning calorimetry, ab initio molecular dynamics

## Abstract

Liquid–liquid phase transition (LLPT) is a transition from one liquid state to another with the same composition but distinct structural change, which provides an opportunity to explore the relationships between structural transformation and thermodynamic/kinetic anomalies. Herein the abnormal endothermic LLPT in Pd_43_Ni_20_Cu_27_P_10_ glass-forming liquid was verified and studied by flash differential scanning calorimetry (FDSC) and ab initio molecular dynamics (AIMD) simulations. The results show that the change of the atomic local structure of the atoms around the Cu-P bond leads to the change in the number of specific clusters <0 2 8 0> and <1 2 5 3>, which leads to the change in the liquid structure. Our findings reveal the structural mechanisms that induce unusual heat-trapping phenomena in liquids and advance the understanding of LLPT.

## 1. Introduction

Liquid–liquid phase transition (LLPT) has long been postulated to be connected with liquid anomalies in structure, thermophysical properties, and dynamic crossovers, and thus provides an opportunity in elucidating the fundamental relationships between the structure and properties of liquid, which are far from being well understood. Due to the efforts of the researchers in broad research fields, a few LLPTs have been observed in various systems, ranging from unary to multi-component systems and spanning from network-forming to glass-forming liquids, such as Si [1,2,3,4], P [5,6], Ce [7], Sn [8], water [9,10,11], SiO_2_ [12], Ga_50_In_50_ [13], and Al_2_O_3_-Y_2_O_3_ [14,15]. In recent years, LLPT has been found in many metal systems, such as Pd-based [16], Fe-based [17], Zr-based [18,19], and La-based [20] systems. However, at present, the understanding of the formation mechanism of LLPT is still not sufficient. On the one hand, the small heat flow related to LLPT is difficult to detect by the traditional differential scanning calorimetry (DSC), and on the other hand, the evidence of LLPT may be obscured by the nucleation in the undercooled liquid [21].

Pd-Ni-P is a system with an excellent glass-forming ability (GFA) [22], which can be fabricated into a centimeter-sized metallic glass at a cooling rate as low as 0.17 K/s. Lan et al. revealed a hidden amorphous phase and LLPT in a supercooled liquid regime when the metallic glass is heated up to liquid [16]. In our previous work, an LLPT was observed in a superheated liquid regime during the rapid cooling of liquid Pd-Ni-P [23]. This structural transition is quite anomalous since it is endothermic and not beneficial for the enhancement of the short-range order of the structure. This kind of LLPT is contrary to other exothermic LLPTs, and the mechanism remains elusive. Cu is quite similar to Ni, with their radii being 1.28 and 1.24 Å, respectively. It has been illustrated that the similar element substitution could exert marked influence on the structures and properties, such as the glass forming ability [24] and magnetic properties [25]. After the substitution of Ni by the similar element of Cu, Pd-Ni-Cu-P has shown a large GFA, a critical size being over 70 mm [26], and sluggish crystallization kinetics, enabling it as a good choice for observing structural changes [27,28]. It is quite interesting to know whether this similar element substitution could influence the LLPT, which will further deepen our understanding of endothermic LLPT and the fundamental relationships between the structures and properties of the liquid [29,30,31,32,33].

In recent years, many novel methods have been developed to investigate the LLPT, observing the evolution of microstructure in the alloy system through experimental and simulation methods [34,35,36]. Yu et al. found a temperature-induced LLPT in a GaIn alloy through in situ high-energy X-ray diffraction (XRD) and ab initio molecular dynamics (AIMD) simulations [13]. Su et al. found an LLPT in an AuGa alloy through in situ high-energy XRD, extended X-ray absorption fine structure analysis, and AIMD simulations [37]. Recently, rapid scanning chip calorimetry technology has been well-developed in differential scanning calorimetry (DSC) measurement. Mettler-Toledo Company has developed a flash differential scanning calorimeter using chip sensor which is sensitive to small endothermic and exothermic peaks and has played important roles in the experimental verification of high-temperature LLPT [38]. In our previous work, the endothermic LLPT in Pd-Ni-P liquid was verified by flash differential scanning calorimetry (FDSC) and in situ synchronous XRD [23]. Cheng et al. found through FDSC that in a Yb-Zn binary alloy, a dynamic-driven LLPT exists at a temperature above liquidus temperature [38]. Despite many efforts devoted to exploring the structural variations in LLPT, the microscopic details remain elusive, and hence further efforts, especially the combination of FDSC experimental verification and AIMD simulation, are still required to unveil the mystery of LLPT.

In this paper, FDSC measurement of Pd_43_Ni_20_Cu_27_P_10_ metallic glass was carried out under linear heating and ultra-fast cooling. For elucidating the microscopic details of the structural transition, the atomic structure and various structure-sensitive physical properties of liquid Pd_43_Ni_20_Cu_27_P_10_ were investigated by AIMD simulations. The pair distribution function, Voronoi polyhedra, bond order orientation, and bond angle distribution were applied to characterize the atomic structures, and the self-diffusion coefficient was calculated to study the change of atomic dynamics. The results of this work shed further light on the correlations among the structure, thermodynamic properties, and atomic dynamics of glass-forming liquids.

## 2. Experimental and Simulation Details

To prepare the master alloy of Pd_43_Ni_20_Cu_27_P_10_, the mixture of pure Pd (99.99%), Cu (99.99%), and Ni-P pre-alloy (99.99%) was melted in the arc-melting furnace in a high-purity argon atmosphere (see raw materials information in Table S1 in the Supporting Information). For chemical homogeneity, the master alloy was remelted more than five times. Then, the melt-spun ribbons, with a thickness of 20–30 µm and a width of 1 mm, were prepared by a single-roll melt-spinning method under a high-purity argon atmosphere.

The amorphous structure of the melt-spun ribbon samples was characterized by XRD, as shown in Figure S1. The thermal stabilities of amorphous alloys, including glass transition temperature (*T_g_*), Curie temperature (*T_c_*), crystallization onset temperature (*T_x_*), melting temperature (*T_m_*), and liquidus temperature (*T_l_*) were determined by DSC at a heating rate of 0.67 K/s. The qualitative assessment of the content of constituent elements in the Pd_43_Ni_20_Cu_27_P_10_ amorphous alloy was performed using energy dispersive spectroscopy (EDS) (Figure S2). The results of the qualitative analysis confirmed a homogeneous distribution for all component atoms. Figure S3 gives the semi-quantitative atomic percentage of each element in the Pd_43_Ni_20_Cu_27_P_10_ amorphous strip, which is quite close to the desired constitution.

METTLER TOLEDO Flash DSC 2+ (Mettler Toledo, Zurich, Switzerland) with a maximum heating and cooling rate of 40,000 K/s was used during the experiment. FDSC samples were prepared by cutting thin metallic glass ribbons into around 50 × 50 μm^2^ to around 100 × 100 μm^2^ pieces weighing 1–5 μg under a stereo microscope and then transferred using an electrostatic hairbrush to the central area of the chip sensor. The sample was repeatedly heated from room temperature to 900 °C at a heating rate of 500 K/s and then cooled to room temperature with an interval of 1 s. We cycled more than 10 times at different cooling rates (400 K/s, 500 K/s, 800 K/s, 1000 K/s, 1500 K/s, 2000 K/s, and 2100 K/s), during which the sample was continuously purged with pure argon to avoid oxidation.

AIMD simulations were performed by using the Vienna Ab initio Simulation Package (VASP) based on density functional theory (DFT) [39]. The generalized gradient approximation (GGA) with the Perdew–Burke–Ernzerhof (PBE) functional was adopted to describe electron exchange and correlation functions [40]. The projector augmented-wave (PAW) pseudopotentials were used, and for the accuracy of calculation, the energy cutoff was uniformly set to be 520 eV. The canonical ensemble (NVT), namely, constant number of atoms, volume, and temperature, was used, and the Nosé–Hoover thermostat was utilized to adjust the temperature. Only Γ point was applied to sample the Brillouin zone. To integrate Newton’s equations of motion, the velocity Verlet algorithm was used with a timestep of 3 fs.

An initial random structure of 200 atoms (including 86 Pd, 40 Ni, 54 Cu, and 20 P atoms) in a cubic simulation box with periodic boundary conditions was completely melted at 1500 K and relaxed for 5000 timesteps. To obtain the vitreous structure, the melt was quenched to 300 K stepwisely with a cooling rate of 4 × 10^13^ K/s. For every 100 K, the volume of the simulation box was adjusted to ensure that the average total pressure was close to 0 (within ± 0.5 kbar). Then, the system was run for additional 5000 timesteps, and the last 4500 timesteps were used for trajectory production and property analysis.

## 3. Results and Discussion

### 3.1. Experimental Results

**Figure 1:**
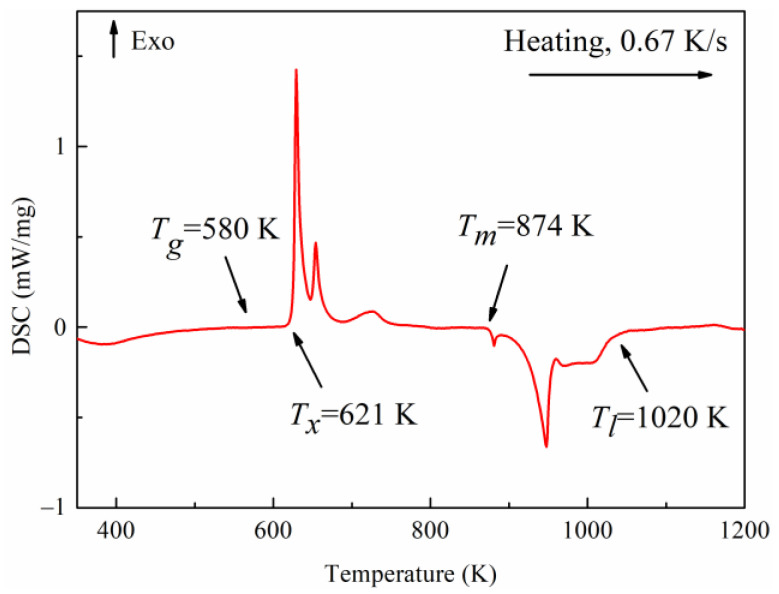
DSC trace of the Pd_43_Ni_20_Cu_27_P_10_ metallic glass measured at a heating rate of 0.67 K/s.

DSC analysis for monitoring changes in thermodynamic properties of the Pd_43_Ni_20_Cu_27_P_10_ spin ribbon was based on the changes of *T_g_*, *T_x_*, *T_m_*, and *T_l_*. As shown in Figure 1, glass transition (endothermic phenomenon) occurs at *T_g_* = 580 K, exothermic crystallization occurs at *T_x_* = 621 K, the melting point is at *T_m_* = 874 K, and the liquidus temperature is at *T_l_* = 1020 K. Consequently, the width (Δ*T_x_* = *T_x_* − *T_g_*) of the supercooled liquid region is 41 K. The measured *T_g_* and *T_x_* are close to those of Pd_43_Ni_10_Cu_27_P_20_ [41] and Pd_42.5_Cu_30_Ni_7.5_P_20_ [42] (both of them have *T_g_* of 568 K and *T_x_* of 640 K). However, the liquidus temperature of the studied Pd_43_Ni_20_Cu_27_P_10_ is much higher than the 850 K of Pd_42.5_Cu_30_Ni_7.5_P_20_.

**Figure 2:**
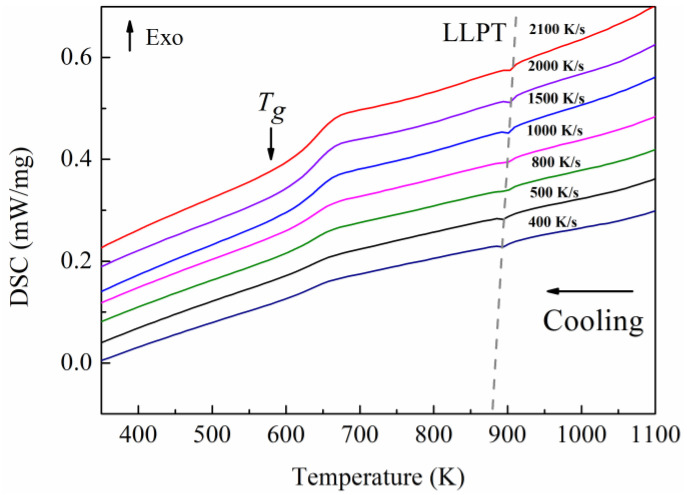
FDSC curves of Pd_43_Ni_20_Cu_27_P_10_ at different cooling rates. The gray dashed line represents the trend of anomalous endothermic peaks.

Although conventional DSC is a very effective tool for exploring the glass transition, the low scan rate limits its application in investigating thermodynamic and dynamic properties. The heat flow versus temperature behavior of Pd_43_Ni_20_Cu_27_P_10_ has been obtained as a function of different cooling rates with the help of FDSC, as illustrated in Figure 2. Intriguingly, an anomalous endothermic peak presents at 893 K for the cooling rate of 400 K/s. The peak temperature shifts to a higher value as the cooling rate increases, and it attains 902 K at the cooling rate of 2100 K/s. The endothermic peak occurring in glass-forming liquid regime is a signature of thermodynamic phase transition, namely, LLPT. The anomalous endothermic LLPT in the Pd-Ni-Cu-P system was also observed in our previous work for Pd_40_Ni_40_P_20_ [23]_._ The atomistic insights into the structural evolution of the transition are discussed below.

### 3.2. AIMD Simulations

To understand the anomalous endothermic peak found in FDSC and the microscopic details of the structural transition, the thermophysical properties, atomic local structure, and dynamics of liquid Pd_43_Ni_20_Cu_27_P_10_ were studied by AIMD simulations.

#### 3.2.1. Thermophysical Properties

**Figure 3:**
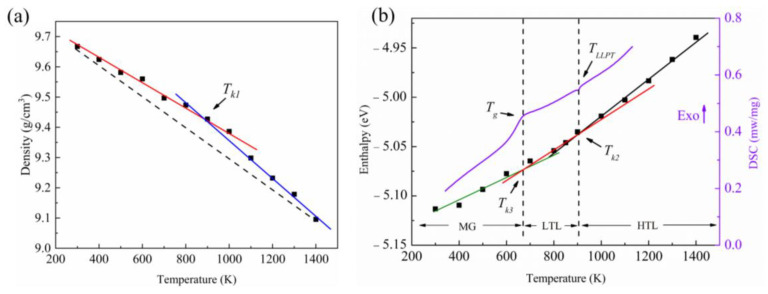
Temperature-dependent variation of (**a**) density and (**b**) enthalpy per atom for Pd_43_Ni_20_Cu_27_P_10_ liquid obtained from the AIMD simulations, where the purple curve denotes the results of the FDSC test at the cooling rate of 2100 K/s shown in Figure 2. The fitting lines are shown together with the dots.

The temperature-dependent variations of density for liquid Pd_43_Ni_20_Cu_27_P_10_ are plotted in Figure 3. A linear fit to AIMD results is applied to express the relation between temperature, *T*, and density, *ρ* (Figure 3a). Clearly, the behavior of the density changes at a temperature *T_k_*_1_ of 890 K. The fitting equations are $\rho =9.785-3.96464\times 10^{-4}T$ (*T* > *T_k_*_1_) and $\rho =10.052-6.7446\times 10^{-4}T$ (*T* < *T_k_*_1_). The variation of the averaged enthalpy as a function of temperature is illustrated in Figure 3b. A kink is also seen at around *T_k_*_2_ = 853 K. At temperatures above *T_k_*_2_, enthalpy, *H*, changes following $H=-5.15098+1.17071\times 10^{-4}T$ and $H=-5.21572+1.95746\times 10^{-4}T$ at temperatures below *T_k_*_2_. Between 600 K and 700 K, a kink can be obviously found if only the data below *T_k_*_2_ are utilized for analysis, and the kink temperature, *T_k_*_3_, is close to the glass transition temperature measured by FDSC. Hence, the temperature regime of the curve can be classified into three parts, separated by *T_k_*_2_ and *T_k_*_3_, namely, high-temperature liquid (HTL) regime, low-temperature liquid (LTL) regime, and metallic glass (MG) regime. The kink temperatures of density, *T_k_*_1_, and enthalpy, *T_k_*_2_, are quite close to the LLPT temperature measured by FDSC. From the continuous change of enthalpy and density, it can be concluded that the LLPT found in this work is a second-order transition, similar to that of Sn [8], in which a continuous change in the order parameter (a measurable quantity that characterizes the phase) is the characteristic as the system undergoes the transition.

#### 3.2.2. Atomic Structures

We now turn our attention to the microscopic details of the structural transition of LLPT. The atomic structure was analyzed with pair distribution function, Voronoi polyhedra, bond order orientation, and bond angle distribution on the data obtained from AIMD simulations.

##### Pair Distribution Function

**Figure 4:**
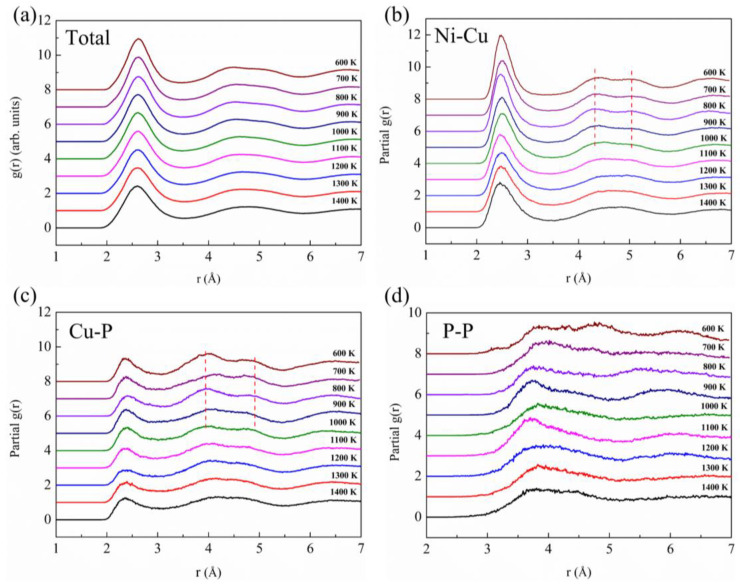
Total and partial PDFs in Pd_43_Ni_20_Cu_27_P_10_ liquid at various temperatures. (**a**) Total, (**b**) Ni-Cu partial, (**c**) Cu-P partial, and (**d**) P-P partial. For clarity, the curve is shifted by 1 successively from the bottom to the top.

**Figure 5:**
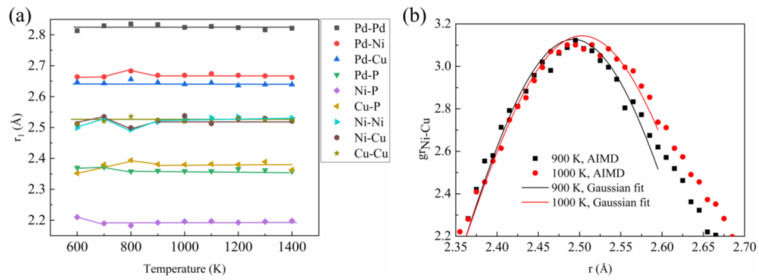
(**a**) Change of the nearest neighbor distance of different atomic pairs with temperature. (**b**) Enlarged view of the first peak of PPDF for Ni-Cu atom pair at 1000 K and 900 K.

The calculated total pair distribution function (PDF) and partial PDFs (PPDFs, *g*_ij_) of liquid Pd_43_Ni_20_Cu_27_P_10_ at different temperatures are displayed in Figure 4. For clarity, only the PPDFs of three atom pairs are shown here since the other seven pairs show similar PPDFs with Ni-Cu and Cu-P. The PPDFs for the other atom pairs are shown in Figure S4. From Figure 4a, it can be seen that the intensity of the first peak of the total PDF, *g*_total_(r), is gradually increased with the decrease of the temperature. At the same time, the second peak begins to split at around 800 K, indicating a certain degree of correlation between the connections and atomic clusters. As indicated by the red dotted lines in Figure 4b–d, the second peak of nine PPDFs, except the *g*_P-P_, begins to split from a single peak into two subpeaks when the liquid is cooled down below around 1000 K. The split of the second peak for P-P pair becomes obvious as the temperature decreases below 700 K. To view the change of the structure, the nearest neighbor distance, *r*_1_, is obtained from a Gaussian peak fitting for the first peak of PPDF, and the results are shown in Figure 5a. For demonstration, the Gaussian peak fitting for the first peak of PPDF for the Ni-Cu atom pair is presented in Figure 5b. Clearly, at temperatures above 900 K, the *r*_1_ for all the atom pairs remains nearly unchanged. However, atom pairs of Ni-Pd, Ni-Ni, Ni-Cu, and Cu-P show apparent variations when the temperature is decreased below 900 K, which is quite close to the LLPT temperature. The *r*_1_ for Ni-Pd and Cu-P increases first and then decreases as the temperature decreases. However, the *r*_1_ for Ni-Ni and Ni-Cu has an obvious abnormal decrease between 1000 K and 900 K. From Figure 5b, it can be seen that the first peak of PPDF for the Ni-Cu atom pair has a noticed left shift at 900 K when compared with the case of 1000 K, indicating that the *r*_1_ of 900 K is smaller than that of 1000 K.

##### Voronoi Polyhedra

**Figure 6:**
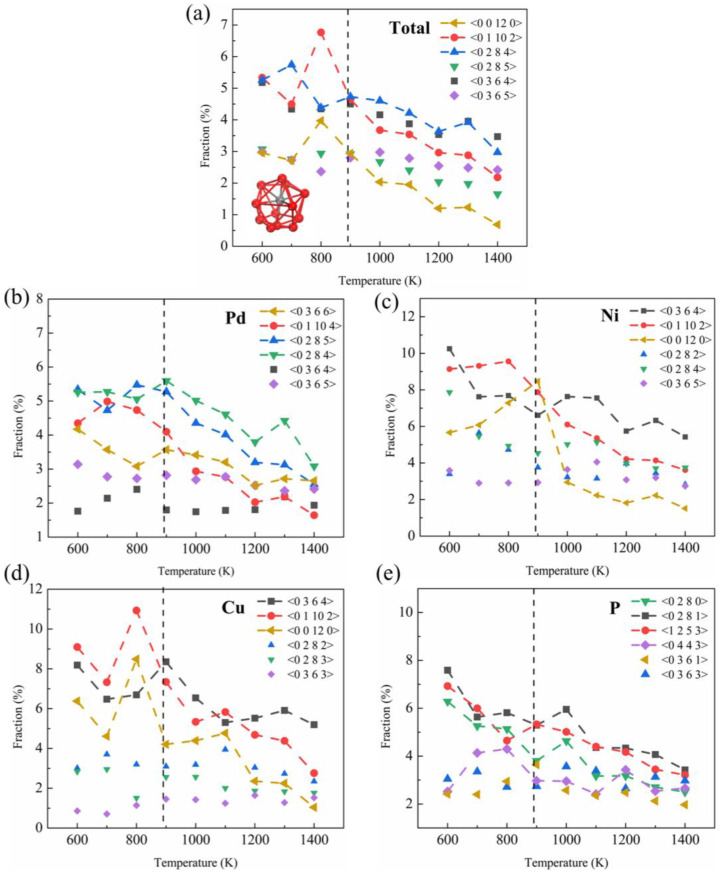
(**a**) Total VP of Pd_43_Ni_20_Cu_27_P_10_ as a function of temperature. The model at the right figure is a typical <0 0 12 0> polygon. (**b**–**e**) Variation of VP centered on Pd, Cu, Ni, and P at different temperatures. The scattering points represent VP with little fractional change over the entire temperature range studied, while the scattering line represents VP with a significant change with temperature. The black dashed line represents the temperature at which LLPT occurs.

To gain further insights into the atomic local structure from a geometrical perspective, the changes in the cluster were analyzed using the Voronoi tessellation method [43]. Voronoi polyhedra (VP) analysis is an efficient method for analyzing local atomic environments and clusters in liquids and glasses. The Voronoi index is expressed as <*n*_3_ *n*_4_ *n*_5_ *n*_6_>, where *n_i_* represents the number of *i*-edged faces. Figure 6 shows the temperature dependence of the fraction of the dominant six VPs centered on Pd, Cu, Ni, and P atoms. It is worth noting that after LLPT, the fraction of the icosahedral-like VP with dense atomic packing, including <0 0 12 0> and <0 1 10 2>, has a marked growth (Figure 6a), and some studies have shown that the icosahedral cluster is the main factor for the formation of metallic glass. VP, such as face-centered cubic (fcc), is easy to form around Pd atoms. The population fraction of Cu- and Ni-centered <0 0 12 0> and <0 1 10 2> (Figure 6c,d), corresponding to icosahedral and twisted icosahedral clusters, respectively, has an obvious increase after LLPT, whereas the <0 3 6 4> fcc cluster is significantly reduced. In the P-centered cluster, the double-top square anti-prismatic clusters of <0 2 8 0> and <1 2 5 3> are significantly reduced, which also shows that LLPT is not conducive to <0 2 8 0> and < 1 2 5 3> cluster increase. The results of VP also show that LLPT promotes the formation of five-fold symmetry to a certain extent.

##### Bond Order Orientation

**Figure 7:**
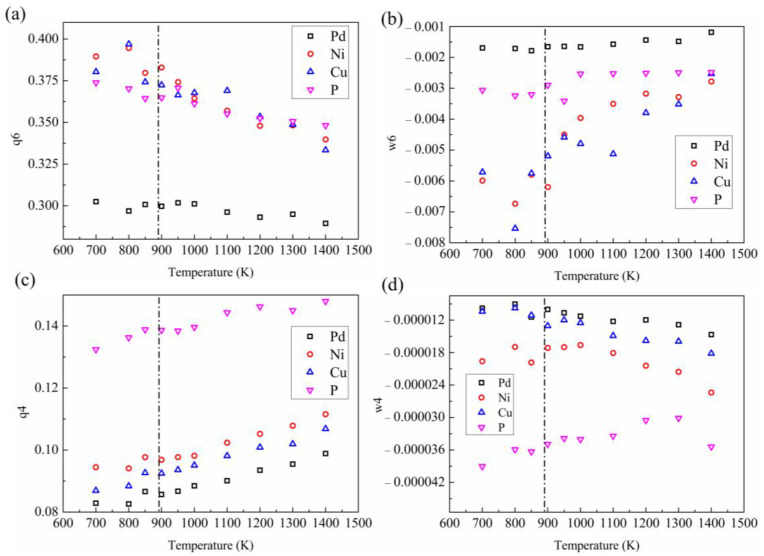
Temperature dependence of BOO parameters *w*_6_ (**a**), *q*_6_ (**b**), *w*_4_ (**c**), and *q*_4_ (**d**) of each element. The black dashed line represents the temperature at which LLPT occurs.

To find the potential change in local structure, we analyzed the bond order orientation (BOO) of the system based on the spherical harmonic function [44]. The spherical harmonic function is a quantitative description of the symmetry of different bond orientations around the central atom. BOO analysis can sensitively reflect the changes in local structure. The average BOO parameters are used to judge the structural difference between high-temperature and low-temperature liquids. We first calculated the BOO parameters of *q*_4_, *q*_6_, *w*_4_, and *w*_6_ of each atom during the cooling process. Figure 7a–d shows the change of average *q*_6_, *w*_6_, *q*_4_, and *w*_4_ at different temperatures. It can be seen that as the temperature decreases, the *q*_6_ value of Ni, Cu, and P element increases. In the process of transforming high-temperature liquid into low-temperature liquid, only the increasing trend of *w*_6_ in P element is slowed down (Figure 7b). Immediately after LLPT, as indicated by the black dotted line, the decrease of *w*_6_ for P element is most prominent, and the other elements are not obvious. In *w*_4_ and *q*_4_, a similar phenomenon also exists. The BOO of P element has an inflection point near LLPT, and the trend of BOO for other elements does not change much. This indicates that the P element has an obvious change in the local environment and plays a key role in LLPT.

##### Bond Angle Distribution

**Figure 8:**
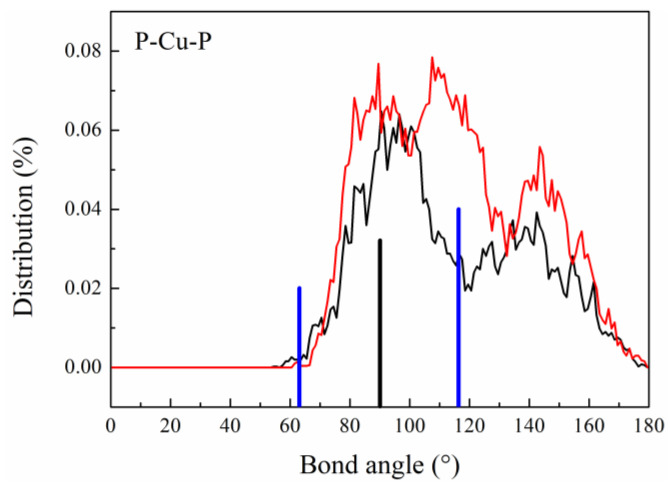
BADs of P-Cu-P in Pd_43_Ni_20_Cu_27_P_10_ liquid at different temperatures, where the black curve represents 1200 K and the red curve denotes 700 K. The blue vertical line corresponds to the characteristic angle of the icosahedral clusters, and the black vertical line corresponds to the characteristic angle of the octahedral ones.

To analyze the short-range structure of atoms in the three-dimensional space, we performed a bond angle distribution (BAD) analysis [43], which provides more information on the chemical and topological orderings of the higher-order correlations. The BAD can be obtained by considering a group of three atoms, where one atom is denoted as a central atom, and the other two nearest neighbors are defined as side atoms. The BADs of Pd_43_Ni_20_Cu_27_P_10_ at different temperatures are illustrated in Figure 8. The characteristic angles of ideal icosahedron clusters are 63.4° and 116.6°, and the BADs of P-Cu-P change most obviously after LLPT, from an octahedron structure under high-temperature liquid to an icosahedron structure under low-temperature liquid. The remaining BADs change little before and after LLPT (Figure S5), among which the BADs of Pd-Ni-Pd and Pd-Cu-Pd are in the best agreement with the icosahedral characteristics, which is also consistent with the number of Cu and Ni icosahedron-like clusters in the previous VP. The deviation of other bond angles from the characteristic angles of the icosahedron may be ascribed to the distorted icosahedral cluster composition. In addition, the hump located at a bond angle of 154° in P-Ni-P became clear after LLPT, indicating a change in short-range order. These results further confirm that the structure has changed and that Cu and P atoms play important roles in the LLPT process.

#### 3.2.3. Atomic Dynamics

**Figure 9:**
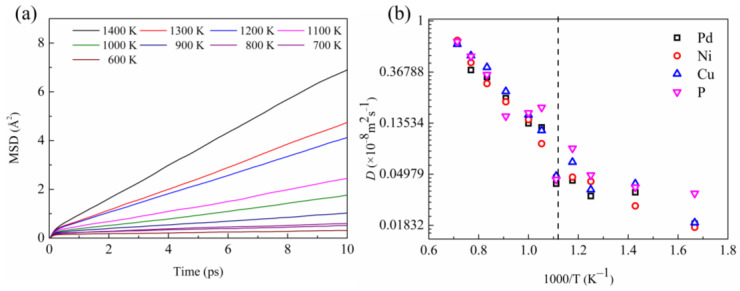
(**a**) Time dependence of the MSD of liquid Pd_43_Ni_20_Cu_27_P_10_ during cooling; (**b**) *D* for Pd, Ni, Cu, and P atoms as a function of temperature in the liquid Pd_43_Ni_20_Cu_27_P_10_. The black dashed line in (**b**) represents the temperature at which LLPT occurs.

In general, the structural transition in metallic liquids also affects their kinetic properties. From the AIMD simulations, the mean square displacement (MSD) for each temperature was calculated and the self-diffusion coefficient (*D*) was derived from the linear regime of the MSD (the results are presented in Figure 9a,b). It can be observed that the self-diffusion coefficients for all atoms kink near LLPT temperature, and the self-diffusion coefficients for P and Cu elements fluctuate the most. This means that the Arrhenius behavior of the self-diffusion coefficient changes at LLPT temperature. This non-Arrhenius behavior of diffusivity indicates a change of atomic dynamics when LLPT occurs.

From the temperature dependence of the self-diffusion coefficient, the activation energy can be derived. In the high-temperature range, the activation energies of Pd, Ni, Cu, and P components are 5.917 kJ/mol, 6.122 kJ/mol, 6.002 kJ/mol, and 5.565 kJ/mol, respectively, while the activation energies decrease after LLPT respectively to 2.981 kJ/mol, 3.013 kJ/mol, 2.774 kJ/mol, and 1.545 kJ/mol. This phenomenon runs counter to common sense. For a homogeneous liquid system, the activation energy at high temperatures should be lower than that at low temperatures. This abnormality in atomic dynamics further indicates that there is a structural change in Pd_43_Ni_20_Cu_27_P_10_ liquid before and after LLPT, and the P and Cu elements have the greatest influence.

## 4. Conclusions

In summary, the LLPT of glass-forming liquid Pd_43_Ni_20_Cu_27_P_10_ was studied by FDSC, which revealed an anomalous endothermic peak around 893 K (cooling rate of 400 K/s). The structural evolution of the liquid Pd_43_Ni_20_Cu_27_P_10_ was further studied by AIMD simulations. The temperature dependence of the density and enthalpy shows a kink near the occurrence temperature of LLPT, indicating that the LLPT of liquid Pd_43_Ni_20_Cu_27_P_10_ is a second-order phase transition. To elucidate the microscopic details of the LLPT, the atomic structure was studied by analyzing the PDF, VP, BOO, and BAD.

At the occurrence of LLPT, the first nearest neighboring distance undergoes an apparent change. The VP centered on the P atom twists after LLPT, with Voronoi clusters accounting for 3.8% and 5.5% of the <0 2 8 0> and <1 2 5 3>, respectively. The changes in BOO and BAD at the occurrence of LLPT promote the formation of quintuple symmetry, and the characteristic angle of the P-Cu-P bond changes from octahedral of high-temperature liquid to icosahedral of low-temperature liquid. Moreover, LLPT affects the kinetics of liquid Pd_43_Ni_20_Cu_27_P_10_, mainly manifested as atomic activation energy. The atomic activation energies of Pd, Ni, Cu, and P after LLPT decrease from 5.917 kJ/mol, 6.122 kJ/mol, 6.002 kJ/mol, and 5.565 kJ/mol to 2.981 kJ/mol, 3.013 kJ/mol, 2.774 kJ/mol, and 1.545 kJ/mol, respectively. The findings in this work deepen our understanding of the relationship among thermodynamics, structure, and kinetics in glass-forming liquids.

## Data Availability

Data are contained within the article.

## Supplementary Materials

The following supporting information can be downloaded at: https://www.mdpi.com/article/10.3390/ma16124353/s1.
