# Supplementary material for: High-Temperature Liquid–Liquid Phase Transition in Glass-Forming Liquid Pd43Ni20Cu27P10
Source: Materials (Basel). 2023 Jun 13;16(12):4353. doi: 10.3390/ma16124353 (PMC10304335; doi:10.3390/ma16124353)
Supplement: Supplementary file 1 [file materials-16-04353-s001.zip › materials-2439055-supplementary.pdf]

High-temperature liquid-liquid phase transition in glass-forming liquid $Pd_{43}Ni_{20}Cu_{27}P_{10}$

Huanyi Zhou[1], Pengfei Yu[2], Xiaoyu Miao[1], Cunjin Peng[1], Lulu Fu[1], Conghui Si[1], Qifang Lu[1], Shunwei Chen[1,*] and Xiujun Han[1,*]

[1]School of Materials Science and Engineering, Qilu University of Technology (Shandong Academy of Sciences), Jinan 250353, China

[2]School of Materials Science and Engineering, Shanghai Jiao Tong University, Shanghai 200240, China

** Correspondence: swchen@qlu.edu.cn (S.C.); xjhan@qlu.edu.cn (X.H.)*

Equipment: The test methods used in this paper include XRD analysis, DSC analysis, and EDS analysis. The instrument used for XRD is the Bruker D8 Advance X-ray diffractometer of Brock Co., LTD. The anode target is Cu-Kα, the wavelength is 0.15406 nm, the operating voltage and current are 40 kV and 30 mA, respectively, and the scanning speed is 6 °min-1. For the DSC test, 20~30 mg alloy strip samples were taken, cut into a uniform square, and placed in an alumina crucible at a heating rate of 0.67 K /s. The equipment model is METTLER TOLEDO TGA/DSC. For EDS, Hitachi FESEM-4800 was used, and the test accelerated voltage was set to 3 kV.

Table S1. Purity and source of the samples used in this work (The samples were used as received without further purification).

Sample	Purity (wt.%)	Source
Pd	99.99	
Cu	99.99	China New Metal Materials
Ni	99.99	Technology Co., Ltd
Ni_2P pre-alloy	99.9 (Ni:19.35 wt.%)	

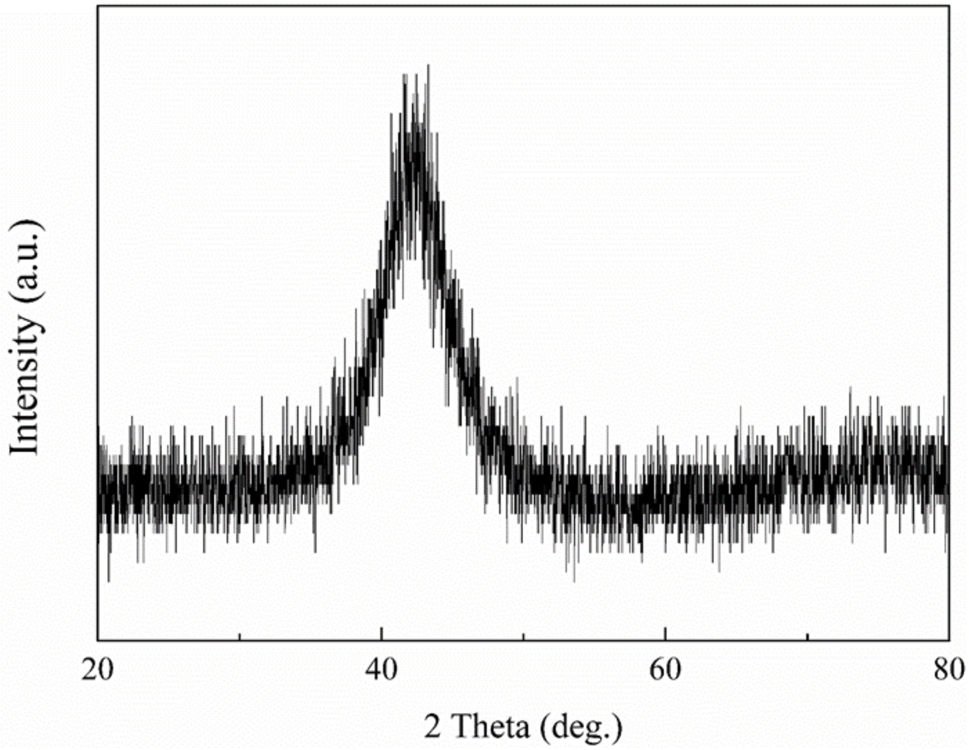

Figure S1. X-ray diffraction (XRD) patterns of the melt-spun $Pd_{43}Ni_{20}Cu_{27}P_{10}$ alloy.

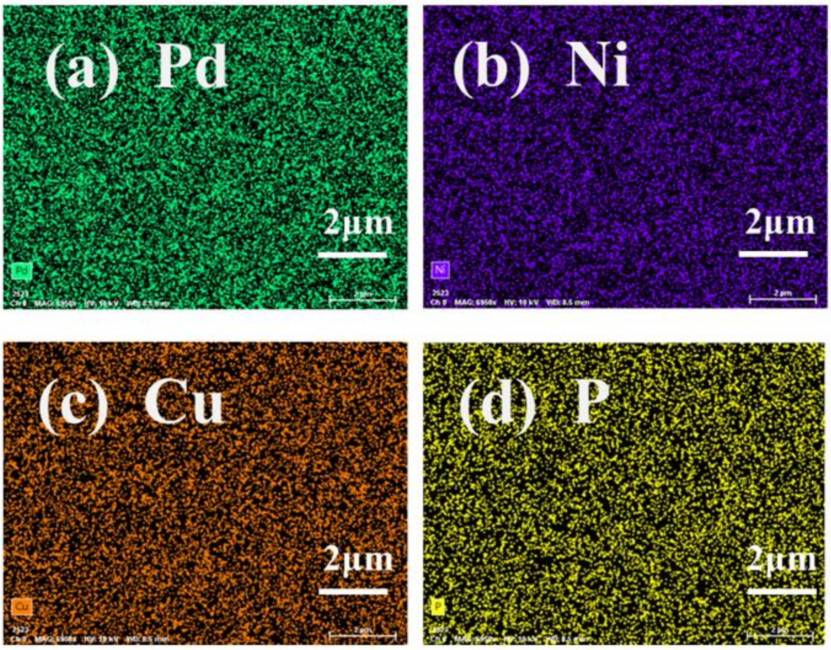

Figure S2. (a)-(d) Energy dispersive spectroscopy (EDS) images for atoms of each component in the $Pd_{43}Ni_{20}Cu_{27}P_{10}$ metallic glass.

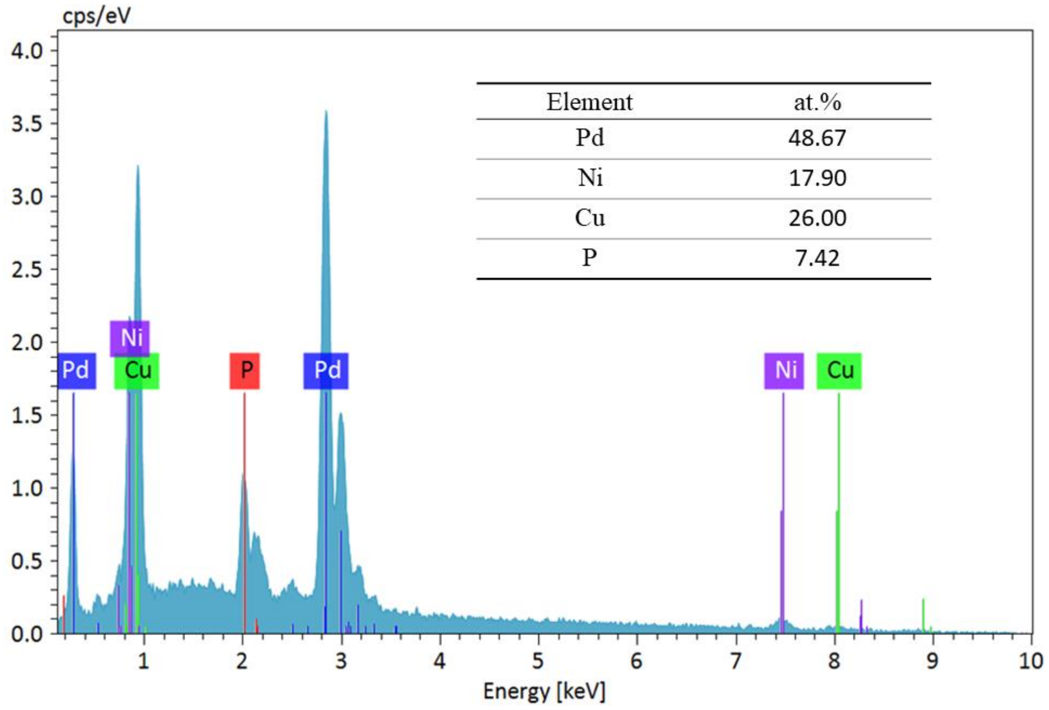

Figure S3. Counts per second (CPS) versus energy for the four component elements of $Pd_{43}Ni_{20}Cu_{27}P_{10}$ metallic glass. The embedded table lists the semi-quantitative atomic ratios of these four elements.

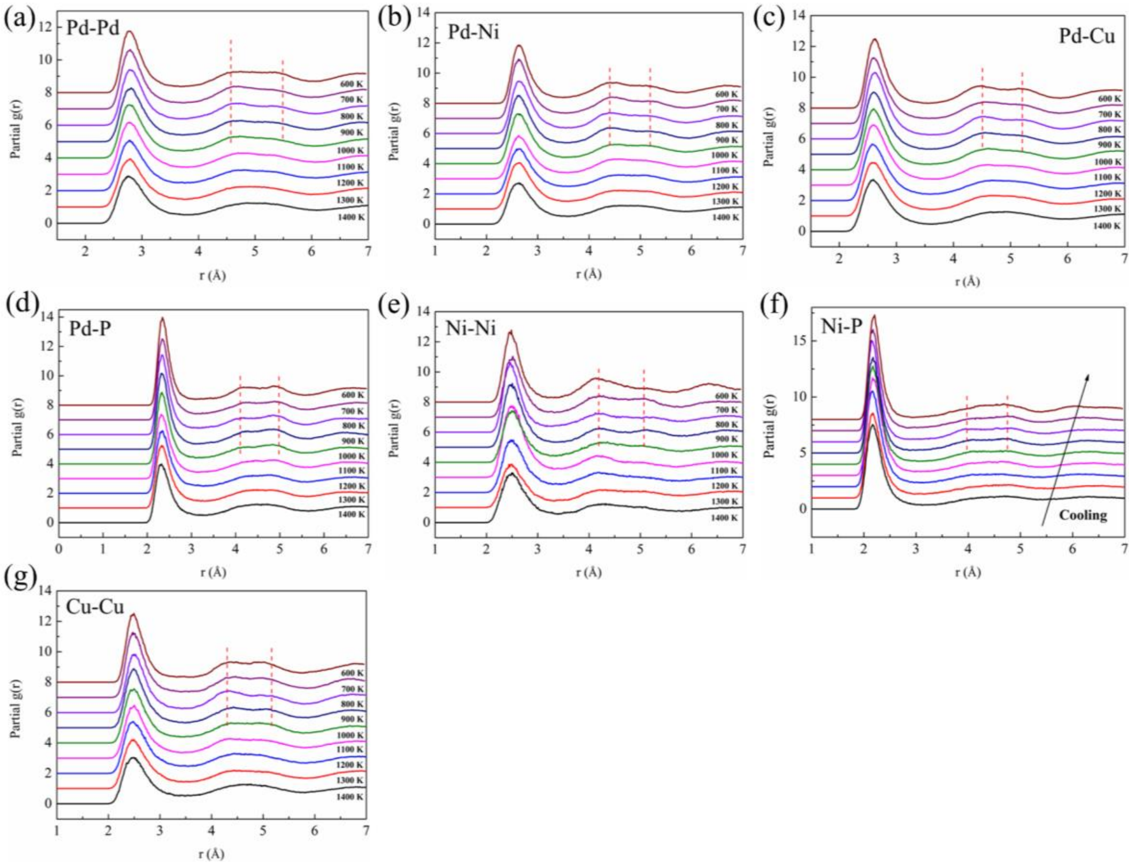

Figure S4. Partial pair distribution function (PDF) in the $Pd_{43}Ni_{20}Cu_{27}P_{10}$ liquid at various temperatures. (a) Pd-Pd partial, (b) Pd-Ni partial, (c) Pd-Cu partial, (d) Pd-P partial, (e) Ni-Ni partial, (f) Ni-P partial, and (g) Cu-Cu partial. For clarity, the curve is shifted by 1 from the bottom to the top.

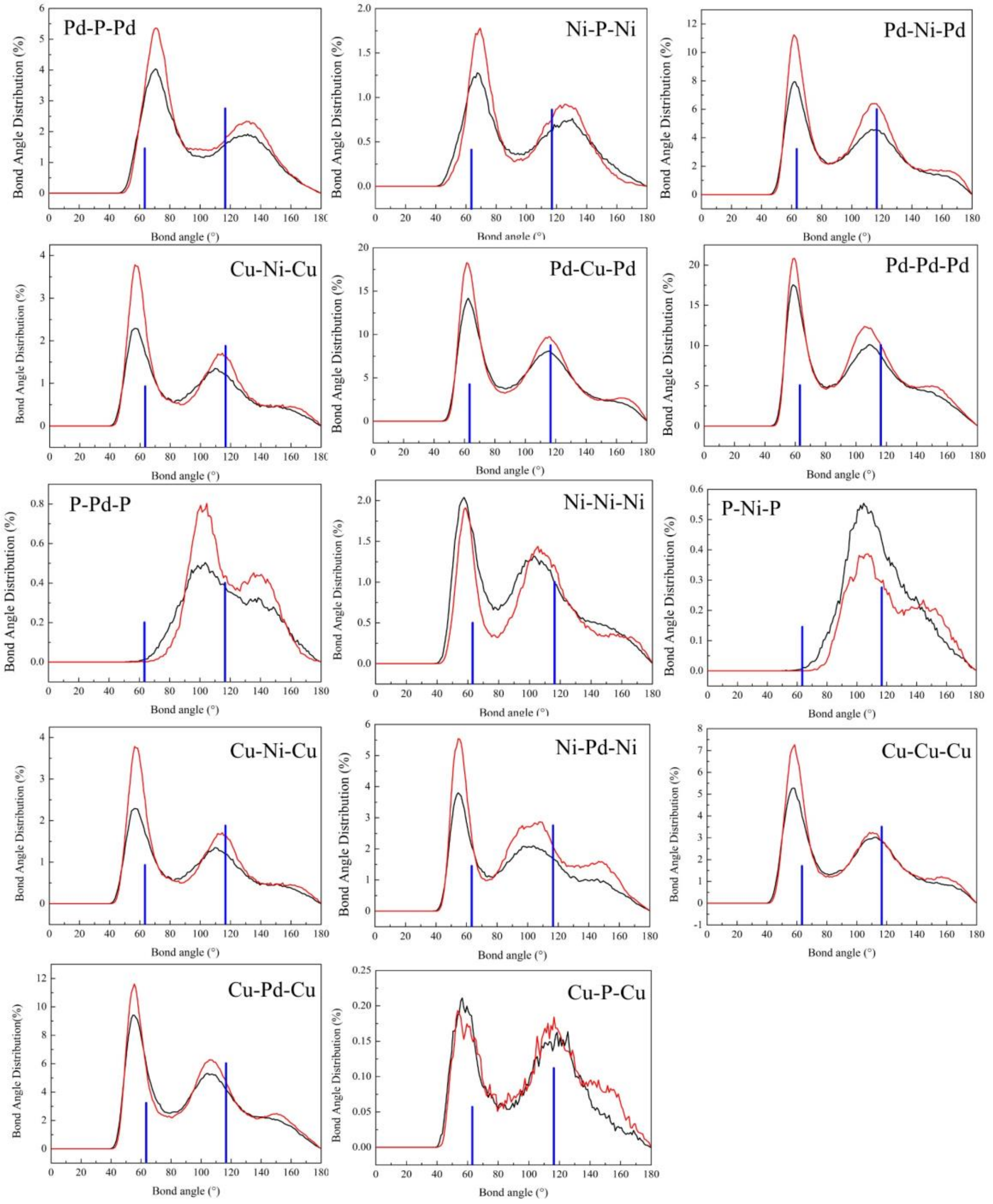

Figure S5. Bond-angle distributions (BADs) in the $Pd_{43}Ni_{20}Cu_{27}P_{10}$ liquid at various temperatures. The black curve represents 1200 K, and the red curve represents 700 K.

Table S2. Cartesian coordinates of the system containing 200 atoms for our AIMD simulations.

Atoms	X	Y	Z
Pd	0.90282	0.11684	0.89217
Pd	0.24423	0.28015	0.56667
Pd	0.10112	0.04311	0.96184
Pd	0.24911	0.32778	0.18779
Pd	0.00025	0.41204	0.11594
Pd	0.48251	0.25888	0.75049
Pd	0.66905	0.08166	0.89086
Pd	0.36459	0.26915	0.91333
Pd	0.21616	0.44759	0.84080
Pd	0.94378	0.55355	0.63451
Pd	0.19874	0.96228	0.84452
Pd	0.18696	0.78178	0.53878
Pd	0.92678	0.37415	0.45635
Pd	0.59889	0.87474	0.37009
Pd	0.68726	0.76590	0.50590
Pd	0.83859	0.30160	0.60761
Pd	0.07784	0.44998	0.33686
Pd	0.28431	0.90143	0.69493
Pd	0.95328	0.30073	0.28125
Pd	0.00822	0.06497	0.61540
Pd	0.78464	0.92702	0.50097
Pd	0.52329	0.45895	0.48177
Pd	0.39032	0.27148	0.43313
Pd	0.10164	0.89110	0.69468
Pd	0.14959	0.06026	0.33195
Pd	0.04519	0.67273	0.95226
Pd	0.69042	0.01828	0.26619
Pd	0.05679	0.22791	0.00026
Pd	0.68273	0.21672	0.61726
Pd	0.92871	0.58258	0.42578
Pd	0.14327	0.96551	0.52773
Pd	0.40269	0.70486	0.05682
Pd	0.08866	0.19871	0.18949
Pd	0.98172	0.57343	0.23464
Pd	0.70840	0.56164	0.89808
Pd	0.66621	0.09890	0.45339
Pd	0.84790	0.73293	0.71436
Pd	0.99151	0.12456	0.43010
Pd	0.46131	0.47573	0.70908
Pd	0.69837	0.15228	0.11800
Pd	0.81102	0.82799	0.32623

Pd	0.12809	0.69123	0.20386
Pd	0.95843	0.75811	0.51846
Pd	0.17298	0.63597	0.81265
Pd	0.50652	0.97403	0.97109
Pd	0.14276	0.54418	0.04458
Pd	0.52935	0.84153	0.71170
Pd	0.96520	0.94826	0.49540
Pd	0.41825	0.67203	0.72500
Pd	0.70209	0.70048	0.14761
Pd	0.42011	0.66504	0.23865
Pd	0.04058	0.44454	0.91436
Pd	0.27591	0.41910	0.03837
Pd	0.51994	0.03898	0.25880
Pd	0.37960	0.45793	0.22905
Pd	0.42053	0.91689	0.14068
Pd	0.58103	0.62592	0.76754
Pd	0.19904	0.09113	0.66194
Pd	0.54303	0.64335	0.58129
Pd	0.43029	0.52278	0.06584
Pd	0.53158	0.34407	0.94300
Pd	0.95771	0.95700	0.83846
Pd	0.88457	0.42779	0.78599
Pd	0.31644	0.09846	0.49948
Pd	0.42379	0.84397	0.32326
Pd	0.69116	0.31435	0.24344
Pd	0.48603	0.78331	0.89377
Pd	0.31631	0.05347	0.95435
Pd	0.98004	0.80359	0.31234
Pd	0.49509	0.14980	0.52876
Pd	0.58901	0.66571	0.01483
Pd	0.64021	0.43063	0.62834
Pd	0.06360	0.43075	0.55954
Pd	0.80881	0.37036	0.11870
Pd	0.70863	0.73185	0.84604
Pd	0.78909	0.83366	0.98638
Pd	0.19967	0.24183	0.80400
Pd	0.71178	0.57334	0.42399
Pd	0.22234	0.51924	0.64644
Pd	0.37662	0.73175	0.54214
Pd	0.89998	0.19019	0.09142
Pd	0.67778	0.39517	0.41929
Pd	0.24064	0.21137	0.03590
Pd	0.72354	0.87701	0.16712
Pd	0.11982	0.67582	0.37659

Pd	0.26459	0.39902	0.38522
Ni	0.79424	0.17993	0.26888
Ni	0.92411	0.89485	0.15073
Ni	0.55117	0.10620	0.67863
Ni	0.86034	0.95175	0.68521
Ni	0.07893	0.61449	0.53437
Ni	0.41204	0.09720	0.11707
Ni	0.45138	0.98213	0.64490
Ni	0.06731	0.09022	0.79356
Ni	0.00240	0.78638	0.81588
Ni	0.96240	0.61151	0.80589
Ni	0.24043	0.02574	0.13780
Ni	0.03484	0.00038	0.13209
Ni	0.61375	0.43512	0.80048
Ni	0.32123	0.73639	0.85392
Ni	0.83977	0.66726	0.02825
Ni	0.25682	0.18616	0.34892
Ni	0.98555	0.00034	0.29711
Ni	0.39852	0.55551	0.39991
Ni	0.73809	0.37567	0.90746
Ni	0.01520	0.24479	0.56126
Ni	0.87812	0.50418	0.96112
Ni	0.21787	0.84852	0.12081
Ni	0.14264	0.13697	0.48739
Ni	0.27494	0.60980	0.50516
Ni	0.96467	0.72613	0.10989
Ni	0.41514	0.29072	0.10497
Ni	0.96669	0.83800	0.98528
Ni	0.35019	0.06264	0.32470
Ni	0.84912	0.09491	0.56205
Ni	0.55334	0.37895	0.13668
Ni	0.84953	0.66851	0.28030
Ni	0.67551	0.96417	0.71510
Ni	0.26559	0.71650	0.68407
Ni	0.68036	0.47075	0.04479
Ni	0.58636	0.19454	0.32832
Ni	0.26494	0.56972	0.29725
Ni	0.12143	0.90111	0.26359
Ni	0.59707	0.96860	0.53155
Ni	0.50736	0.34334	0.29390
Ni	0.24323	0.87789	0.40052
Cu	0.58824	0.00362	0.11171
Cu	0.33383	0.87649	0.97424
Cu	0.27397	0.61248	0.12968

Cu	0.04885	0.72289	0.67419
Cu	0.75463	0.62439	0.58629
Cu	0.53493	0.52775	0.31801
Cu	0.21714	0.72293	0.00454
Cu	0.09773	0.27863	0.42674
Cu	0.56857	0.17012	0.00860
Cu	0.32782	0.12883	0.79217
Cu	0.54261	0.77623	0.17437
Cu	0.56458	0.56586	0.16711
Cu	0.40153	0.43687	0.87162
Cu	0.15918	0.47067	0.19148
Cu	0.63311	0.89713	0.86892
Cu	0.51899	0.83035	0.53262
Cu	0.63754	0.25231	0.80618
Cu	0.27933	0.58388	0.93186
Cu	0.77896	0.53382	0.72359
Cu	0.54563	0.20869	0.16834
Cu	0.79323	0.47021	0.53439
Cu	0.74455	0.99955	0.03076
Cu	0.47386	0.33531	0.59684
Cu	0.84153	0.01221	0.35365
Cu	0.45923	0.99843	0.43327
Cu	0.63034	0.69764	0.30525
Cu	0.86560	0.45699	0.32610
Cu	0.35917	0.43600	0.54474
Cu	0.99761	0.26785	0.82142
Cu	0.78328	0.10307	0.74972
Cu	0.77045	0.20968	0.98708
Cu	0.78660	0.96667	0.84109
Cu	0.70654	0.50104	0.24733
Cu	0.84011	0.53390	0.13133
Cu	0.27334	0.76216	0.26168
Cu	0.43613	0.58220	0.88837
Cu	0.69748	0.78444	0.66312
Cu	0.13576	0.85603	0.95082
Cu	0.91610	0.16728	0.70741
Cu	0.12821	0.34560	0.68244
Cu	0.90664	0.31143	0.95244
Cu	0.36954	0.23889	0.25058
Cu	0.49075	0.13585	0.85786
Cu	0.33282	0.92424	0.52066
Cu	0.82469	0.24879	0.81460
Cu	0.36711	0.18308	0.65162
Cu	0.60507	0.84442	0.02673

Cu	0.90989	0.99388	0.01279
Cu	0.39561	0.95177	0.83038
Cu	0.85195	0.04151	0.16797
Cu	0.06475	0.48201	0.74524
Cu	0.33740	0.33163	0.72547
Cu	0.50215	0.70129	0.39797
Cu	0.81812	0.22802	0.45548
P	0.08850	0.84170	0.41972
P	0.74961	0.36090	0.72823
P	0.53152	0.00025	0.78341
P	0.95335	0.83288	0.66246
P	0.82700	0.71579	0.42441
P	0.88485	0.75077	0.89246
P	0.16341	0.51569	0.46460
P	0.15446	0.33059	0.94479
P	0.19598	0.79641	0.78749
P	0.09084	0.19018	0.68224
P	0.55927	0.51874	0.93510
P	0.29230	0.92448	0.24351
P	0.24845	0.15811	0.20449
P	0.94523	0.14614	0.24872
P	0.71409	0.06631	0.61031
P	0.98241	0.57227	0.05416
P	0.55389	0.28930	0.44551
P	0.83396	0.78087	0.16485
P	0.06684	0.84256	0.12139
P	0.67335	0.32416	0.05391
